# Single-Entity Resolution Single-Cell Nanosensor Reveals Reactive Oxygen Species at Stress Granules Are Formed by Interfacial Redox Chemistry

**DOI:** 10.1021/jacs.5c09338

**Published:** 2025-07-22

**Authors:** Hui Gu, Chaoyi Gu, Andre Du Toit, Wen Yu, Michael W. Chen, Heather L. Struckman, Jonathan R. Silva, Yifan Dai, Andrew G. Ewing

**Affiliations:** † Department of Chemistry and Chemical Engineering, Hunan University of Science and Technology, Xiangtan 411201, China; ‡ Department of Chemistry and Molecular Biology, 3570 University of Gothenburg, Gothenburg 41390, Sweden; § Department of Biomedical Engineering and Center for Biomolecular Condensates, 7548 Washington University in St. Louis, Saint Louis 63130, United States

## Abstract

The electrochemical activities of biomolecular condensates represent a new fundamental functioning mechanism in biochemistry and cell biology. However, our understanding of the underlying molecular mechanism and the interfacial field-dependent chemical activities remains limited. This is due to the lack of technology to probe such activities in real time and at a single-condensate level. Stress granules (SGs) are membraneless organelles that form in the cytoplasm to adapt to cell stress, which are found to encapsulate reactive oxygen species (ROS) in our lab. Here, we design and implement a collision-based electrochemical nanosensor that enables probing of the redox activities of SGs at a single-condensate level in live cells. We show that ex-vivo separated SGs drive the redox reactions depending on their own interfacial potentials and the constituents of the solution system. Surprisingly, we found that water molecules, rather than solvated oxygen (the main source of ROS produced by a conventional enzyme reaction in cells), are the main chemical origin of the redox activity of SGs. Finally, we demonstrate the application of this electrochemical nanosensor in real-time probing of the generation of hydrogen peroxide from SGs in mammalian cells and show that the electrochemical environment of the cells can regulate the redox activity of SGs. This work uncovers the likely mechanisms encoding nonenzymatic redox activities of SGs and demonstrates a key fundamental technological capability that can be highly useful in exploring the intracellular electroactive pathways of macroscale assemblies.

## Graphical abstract

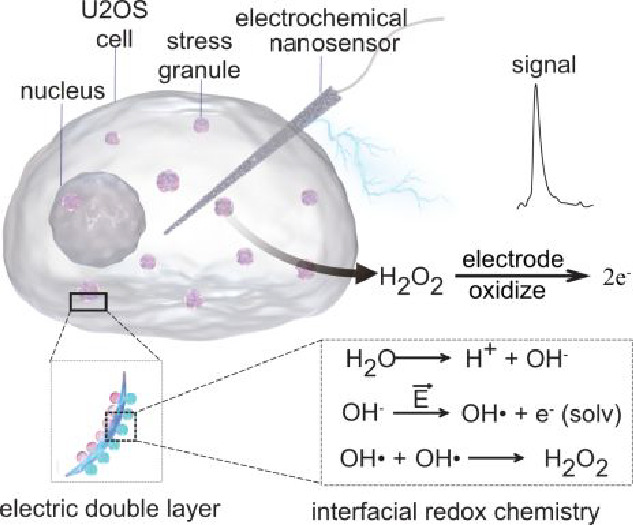

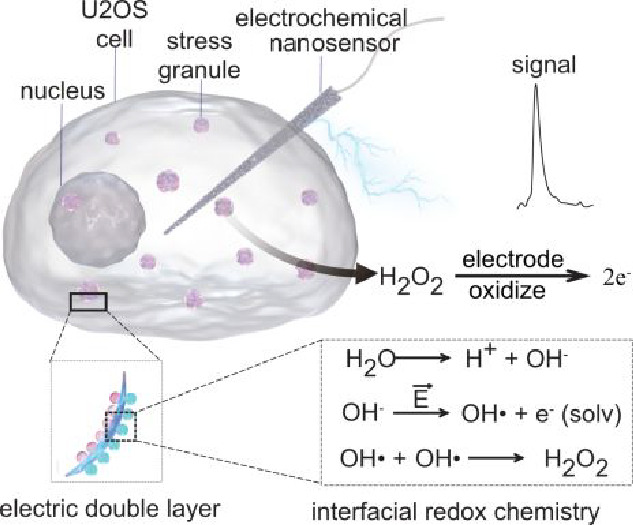

## Introduction

The electrochemical environment within a cell serves as the fundamental physiochemical basis that defines diverse cellular activities.^1,2^ For example, intracellular ion gradients modulate ribosome activity^3^ and initiation of electron transfer powers aerobic respiration.^4^ The current understanding of the electrochemical driving force mostly arises from the studies of membrane ion transporters,^5–10^ which serve as the regulators modulating the electrochemical equilibrium between the intracellular and extracellular spaces. However, in the intracellular space, the general mechanisms that define and regulate global electrochemical environments (i.e., the generation of electroactive species and the electrochemical potential equilibrium) are largely unknown.

Recent works on macromolecular condensation, such as the phase transition of associative protein and RNA molecules, shed light on potential mechanisms governing the intracellular electrochemical activities. The condensation of biomolecules leads to the formation of biomolecular condensates (the dense phase) and a corresponding dilute phase, which collectively regulate the spatiotemporal chemistry of a diverse range of cellular processes from transcription to stress responses.^11–17^ This condensation process is a solvent-mediated phase transition of the whole solution system,^18^ in which a density gradient is established between the dilute and the dense phases across the solution system. Thus, both the biomolecules that drive phase transition and the ions and water molecules that undergo associative or segregated phase transitions possess a distinct concentration between the dilute and the dense phases.^19–22^ This aspect of macromolecular condensation defines its ability to regulate intracellular electrochemical activities. For example, a water concentration gradient defined by biomolecular condensates can regulate the water potential of a cell for osmotic responses.^23^ Intracellular ion gradient modulated by condensate formation can adjust the hyperpolarization of membrane potentials of a cell.^24^

A key electrochemical feature of condensates that has been uncovered recently is the interfacial electric field at the liquid–liquid interface of the condensates,^21^ similar to the electric field at the liquid–air or liquid–solid interfaces.^25–30^ For condensates, this is mediated by the asymmetric ion gradient across the dilute and the dense phases,^20,31–35^ which in turn defines an interfacial electric potential gradient.^21^ As such, the large potential gradient across the dilute and the dense phases, at which charge separation occurs,^20,36^ dictates an interfacial electric field at the surface of condensates.^21,37–41^ As an electric field can substantially lower the reaction barrier of the intermediate state of reactants for diverse chemical reactions,^42,43^ this inherent electric field of condensates can serve as a driving force for chemical reactions, such as the spontaneous generation of hydrogen peroxide (H_2_O_2_) by the redox capability defined by the condensate electric field.^21^ Reactive oxygen species (ROS) such as H_2_O_2_, are critical transient signaling molecules modulating cellular homeostasis and their upregulations or downregulations can lead to diverse sets of diseases, such as neurodegenerative disorders.^44–47^ However, a study of these features in the case of native condensates is currently lacking, and the molecular mechanisms are largely unexplored.

Most of the previous work utilized conventional fluorogenic tools, which took advantage of the irreversible nature of chemical reactions to probe electrochemical activities of condensates.^21^ In contrast, electrochemical approaches enable condensates to be analyzed in real time through single-collision events.

Such collision events between condensates and electrodes represent the interaction between two charged interfaces, thus allowing us to directly probe the interfacial activity of condensates. With electrochemical approaches, we recently discovered that ex-vivo separated SGs contain ROS,^48^ and SGs in the presence of transmitter vesicles cause a fraction of the latter to undergo homotypic fusion.^49^ Stress granules (SGs) are a subset of native condensates, which alter the composition and concentration of cytoplasmic proteins and are believed to play a key role in the pathophysiology of multiple neurodegenerative diseases (NDs).^50–54^ However, it remains unclear whether the origin of the ROS formed within SGs depends on their interface-dependent nonenzymatic redox activity. To identify this, a sensing technology that is capable of differentiating H_2_O_2_ from ONOO^–^, NO·, and NO_2_^–^ is necessary because ONOO^–^, NO·, and NO_2_^–^ are ubiquitously imbricate in the production of ROS if the conventional enzymatic pathway dominates (Figure 1a).^55–57^ The previously developed electrochemical sensor^48^ had insufficient electrochemical resolution to differentiate the four primary ROS/reactive nitrogen species (RNS) owing to the large area of Pt black aggregates. Thus, only H_2_O_2_ could be determined, nor was it applicable in in situ measurements in living cells. Both features can be critical to understanding the mechanism and the origin of the electrochemical activities of SGs.

**Figure 1:**
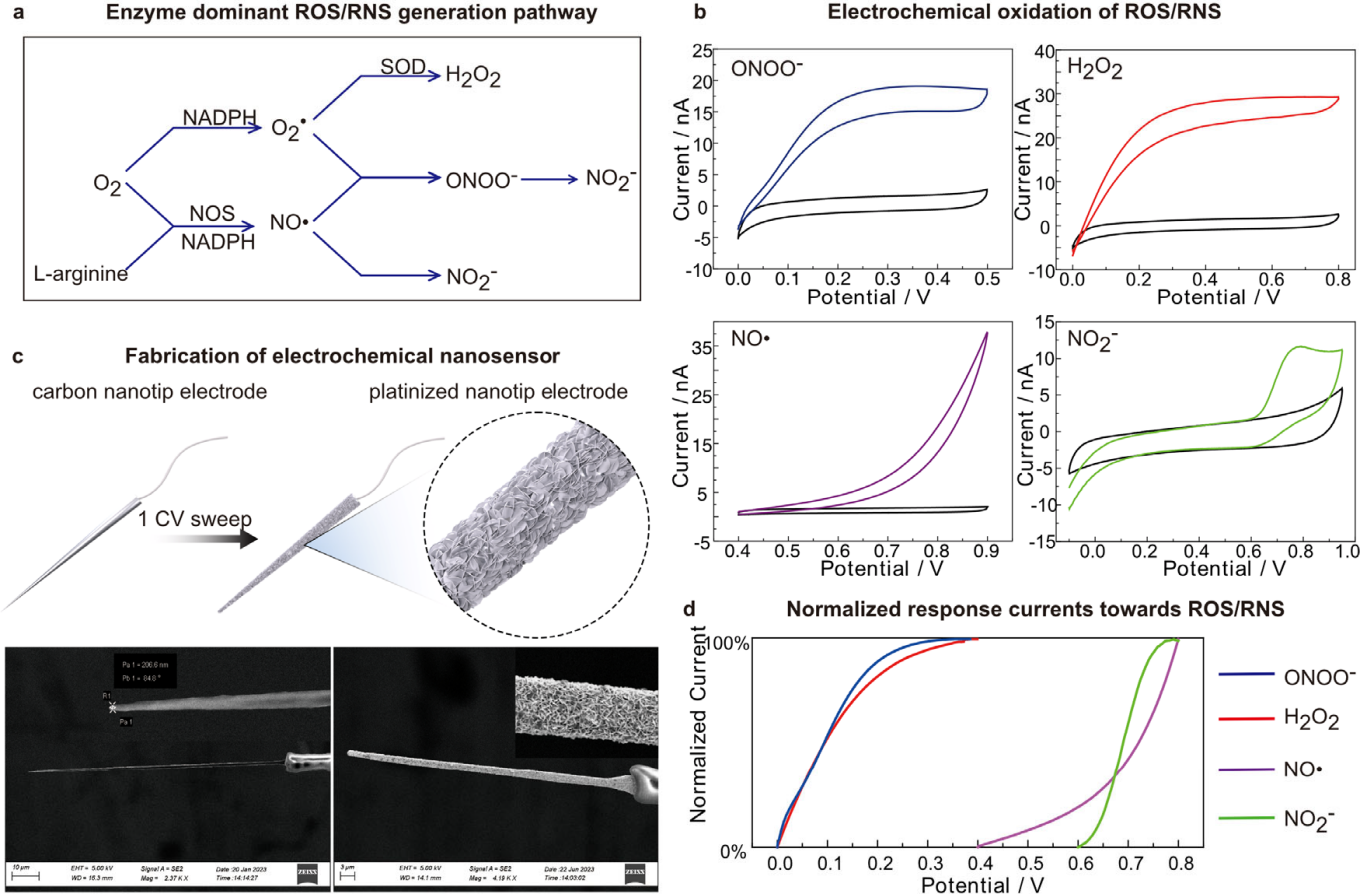
(a) Mechanism of the enzyme-dominant ROS/RNS generation pathway. (b) Electrochemical oxidation of different ROS/RNS. Voltammetric oxidation of ONOO^—^ (pH = 10.0, 10 μM), H_2_O_2_ (pH = 7.4, 10 μM), NO· (pH = 7.4, 10 μM), and NO_2_^—^ (pH = 7.4, 10 μM) in phosphate buffer saline (0.01 M), scan rate 0.1 V/s. (c) Scheme illustrates the fabrication of a nanosensor, and SEM images of flame-etched carbon-fiber nanotip electrodes before and after electrochemical deposition. Inset: magnified SEM images. (d) Normalized currents to compare the responses of the nanosensors toward ONOO^–^, H_2_O_2,_ NO·, and NO_2_^–^.

In this article, we designed an electrochemical nanosensor with the required selectivity to differentiate distinct electrochemical signals of ROS/RNS, thereby allowing us to selectively evaluate the mechanism and the source of the generation of ROS in SGs. This approach utilized a single-particle collision-based detection mechanism in which the electrochemical signal is generated only upon collision with a particle containing active electrochemically active species. This nanotechnology enables the study of the effect of solvent on the redox activities of SGs with single-entity resolution and without interference from the background solvent conditions. We then applied this technology to study the chemical dynamics of the electrochemical activities of native SGs in living cells in real time. The chemical origins and mechanisms discovered in this study suggest the existence of a nonenzymatic pathway for active redox reactions in living cells. These measurements open a new paradigm for understanding cellular electrochemical pathways and reveal a new function of SGs in living cells.

## Results and Discussion

### Design and Engineering of Electrochemical Nanosensors To Quantitatively Differentiate ROS/RNS

To determine the mechanism of ROS generation in SGs, we began by designing an electrochemical nanosensor with sensitivity and selectivity to quantitatively differentiate ROS/RNS with submillisecond resolution. Flame-etched carbon-fiber nanotip electrodes enable insertion through the membranes of living cells while retaining their integrity and allow carrying out electrochemical measurements with increased sensitivity, kinetics, and signal-to-noise ratio and rapid time response.^58,59^ To quantify ROS/RNS, electrochemical deposition of platinum black on nanotip electrodes is a common choice; however, controlling this process during fabrication is challenging, making it difficult to achieve a unique platinum morphology with the necessary electrochemical resolution to differentiate between various ROS/RNS, as well as a time resolution as low as milliseconds.^55,60–63^

It has been reported that a low scan rate during the electrochemical deposition tends to produce larger, more uniform Pt grains, resulting in smoother and denser films.^57^ This can affect the catalytic activity, as the surface structure plays a crucial role in catalytic performance. Inspired by this, we used a different scan rate by sweeping the nanotip electrode for one cycle of cyclic voltammetry from 0 to −500 mV vs Ag/AgCl for electrochemical deposition of platinum on the nanotip electrode (Figure S1a). As a result, we found that decreasing the scan rate as low as 2 mV/s resulted in nanosensors with high catalytic capability to detect and differentiate oxidation waves of ROS/RNS, including ONOO^–^, H_2_O_2_, NO·, and NO_2_^–^ (Figure 1b).

This is comparable to that of Pt nanostructures deposited by a complex chemical process.^55^ Scanning electron microscopy (SEM) was used to observe controllable, well-grown, high-density contiguous platinum particles on the carbon surface (Figure 1c). Voltammograms of ONOO^–^, H_2_O_2_, and NO_2_^–^ were obtained with plateau currents at anodic potentials where the oxidations of ONOO^–^, H_2_O_2_ (*E*_T_ ≥ 300 mV), and NO_2_^–^ (*E*_T_ ≥ 800 mV) on the nanosensor occurred at its maximum rate (Figure 1d). The oxidation current of H_2_O_2_ overlaps with that of ONOO^–^ but can be distinguished from those of NO and NO_2_^–^. The voltametric waves shown in Figure 1d provide a reference set of normalized oxidation voltammograms for each ROS/RNS, illustrating that a detection potential of +400 mV versus Ag/AgCl can be selected for ONOO^–^, H_2_O_2_. The nanosensor demonstrates a linear correlation between current and concentration in the micromolar range (Figure S1b), qualifying it to implement quantitative analysis of ROS/RNS for the analysis of SGs.

### Quantitative Measurement of ROS/RNS at Intracellular SGs

We then penetrated the nanosensor through the membrane of U2OS cells to explore the generation of ROS/RNS by SGs in living U2OS cells in real time (Figure 2a). An electrochemical potential of +800 mV (vs Ag/AgCl) was applied, enabling all four ROS/RNS to be detected. A series of electrochemical measurements in U2OS cells were conducted to confirm the robustness of the nanosensor applied for intracellular analysis of SGs. First, a background amperometric trace without any current spikes was observed in U2OS cells (Figure 2b) before treating with arsenite to induce SG assembly (Figure S2a). This allowed us to exclude the potential interference from the background electroactive species generated by other organelles, which also indicated that the interference from the free condensates is negligibly small. After U2OS cells were treated with arsenite to trigger the formation of SGs (Figure S2b),^64,65^ an amperometric trace with a sequence of spikes was obtained owing to the oxidation of the electroactive species generated by SGs (Figure 2b). To further confirm that the signal is generated from SG collision with the electrode, 1,6-hexanediol, a hydrophobic condensate disrupter which is reported to dissolve the SGs,^66^ was added into the arsenite-treated U2OS cells for 5 min, resulting in a significantly reduced number of current peaks (Figure 2b).

**Figure 2:**
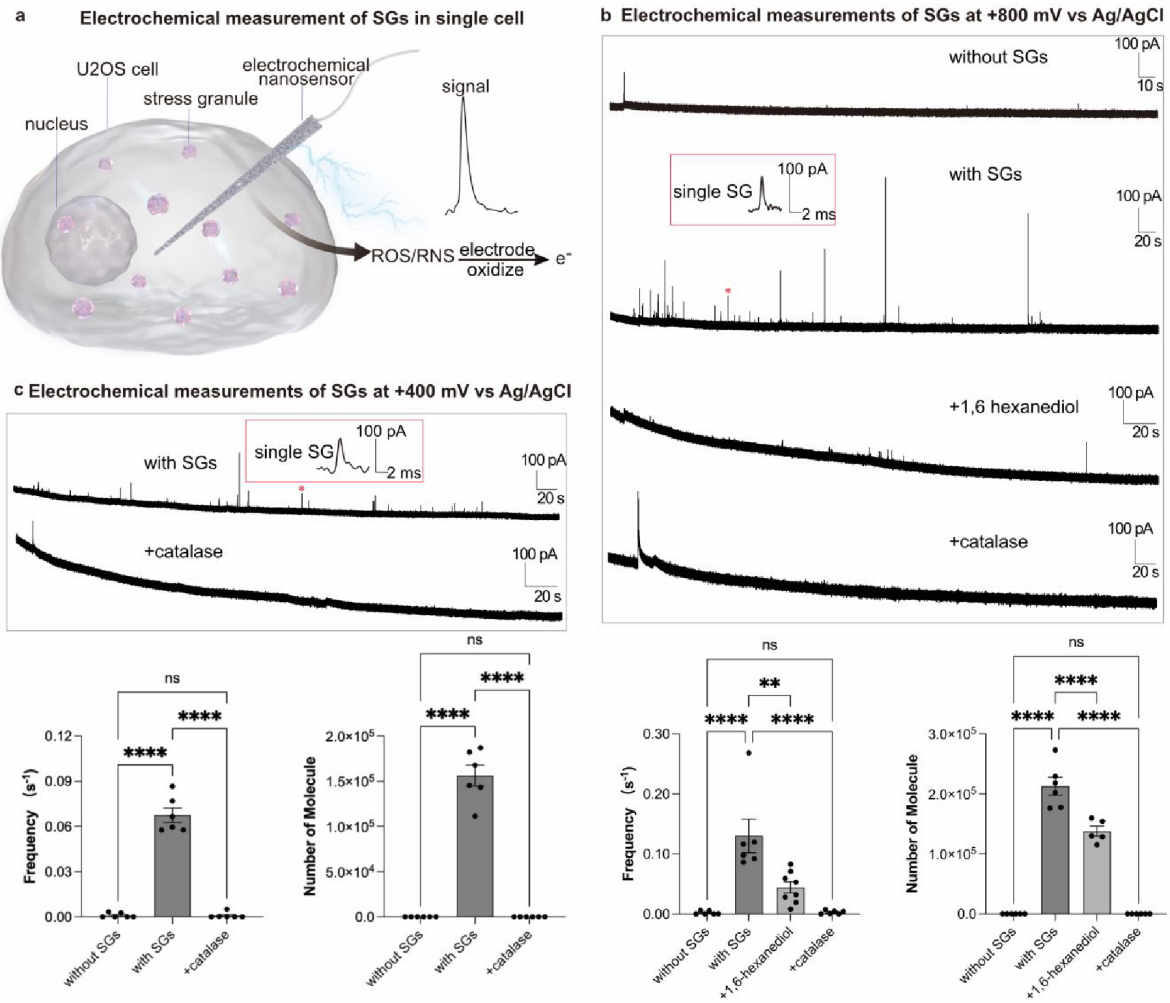
(a) Scheme showing the nanosensor for intracellular quantitative measurement of the ROS/RNS in SGs. (b) Typical amperometry traces obtained by the nanosensors in U2OS cells before or after treatment with 200 μM arsenite (to form SGs) and after treatment with 200 μM arsenite followed treatment by 6% 1,6-hexanediol or by 2.5% catalase-polyethylene glycol (to remove ROS). The potential applied at the nanosensors was +800 mV vs Ag/AgCl. The inset shows amplification of the spike labeled with a red asterisk. Every spike represents a single-SG-detection event. Bar graphs show the statistical median for the frequency of detected SGs events and the number of H_2_O_2_ included in SGs. Statistical data for each group: without SGs, *n* = 6 for 4 spikes; with SGs, *n* = 6 for 391 SGs; +1,6-hexanediol, *n* = 8 for 234 SGs; +catalase, *n* = 6 for 6 spikes. (c) Typical amperometry traces obtained by nanosensors in U2OS cells after treatment with 200 μM arsenite or followed by treatment with 2.5% catalase. The potential applied at the nanosensors was +400 mV vs Ag/AgCl. Bar graphs show the statistical median for frequency of detected SG events and the number of ROS/RNS included in SGs. Statistical data for each group: without SGs, *n* = 6 for 5 spikes; with SGs, *n* = 6 for 196 SGs; +catalase, *n* = 6 for 5 spikes. Traces with more than 15 detected SGs were considered to calculate the median of molecule number. Data represent means ± SEM. One-way ANOVA on ranks: none, *p* > 0.5; **p < 0.01 and ****p < 0.0001.

To distinguish the four ROS/RNS, a potential of +400 mV was applied to the nanosensor to quantitatively oxidize only ONOO^–^ and H_2_O_2_. In this case, a series of current spikes was found only after treating cells with arsenite (Figure 2c). This suggests that the species observed are either H_2_O_2,_ ONOO^–^, or both. In order to distinguish H_2_O_2_ from the other ROS/RNS, arsenite-treated U2OS cells were incubated with a scavenger of H_2_O_2_ (catalase-polyethylene glycol, CAT),^55^ followed by penetration with a nanosensor to measure SGs at +800 mV and +400 mV. No spikes were observed (Figure 2b,c), confirming that the H_2_O_2_ is the main electroactive species in SGs, whereas the other three species (ONOO^–^, NO·, and NO_2_^–^) are not the sources of the electrochemical signal generated by SGs. According to Faraday’s law, *Q* = *nNF*, where *Q* is the charge passed for each spike peak, F is the Faraday constant (96485 coulombs per mole of electrons), n is the number of electrons (*n* = 2 for H_2_O_2_), and *N* is the number of moles oxidized, H_2_O_2_ generated in SGs can be calculated.^49^ It is worth noting that, with consecutive electrochemical measurements both at 400 and 800 mV vs Ag/AgCl, the number of spikes being detected gradually decreases, indicating that the number of single SG collision events per cell decreases over time, as shown in Figure S3. This decrease of frequency might be because of the rapid depletion of the SGs surrounding the nanosensor or the fouling of the nanosensor surface by attachment of the protein and RNA encapsulated in SGs onto the electrode after their collision. However, the H_2_O_2_ number from SGs that collide early is not different from that of SGs that collide late, when there are far fewer events per unit time. Interestingly, when we compared the H_2_O_2_ molecule number obtained at nanosensors polarized at +400 and +800 mV, an obviously higher number and frequency of detected SG events were found at a higher potential. Considering only H_2_O_2_ was found in SGs and our detection technology only analyzed the signal from a single-collision event, this suggests that the interfacial electric field of SGs might be responsible for the redox activity of SGs.^21,38^ This inspired us to test if the generation of H_2_O_2_ by SGs might be dominated not by the classical enzyme involved pathway (Figure 1a) but by a nonenzymatic pathway that is governed by the electrochemical properties of SGs.

### Chemical Origins of H_2_O_2_ Production through a Nonenzymatic Pathway

A key distinction between interfacial electric field dependent redox activities and enzyme-mediated redox activities is the chemical origin (Figure 3a). The conventional enzymatic pathway to produce H_2_O_2_ depends on solvated oxygen molecules,^67^ which requires the transformation of the oxygen molecule into superoxide anion (Figure 1a). Biomolecular condensate-dependent redox activities can simply undergo oxidation of hydroxyl ions into hydroxyl radicals driven by their interfacial electric field,^21,25,26,68,69^ which can directly recombine into the H_2_O_2_ (Figure 3a). Previously, we visualized SGs in U2OS cells and MCF-10A cells by fluorescence images and electrochemical measurements of their ex-vivo separated SGs.^42^ Here, we used ex-vivo separated SGs from arsenite-stressed cells to further investigate the effect of varied solvent environments on their capability to generate ROS by our proposed nanosensors. First, we excluded ONOO^–^, NO·, and NO_2_^–^ contained in ex-vivo separated SGs (Figure S4). As shown, current transients were observed only in solution containing SGs but reduced significantly after the addition of 1,6-hexanediol, and the peaks disappeared after the addition of catalase. A fluorogenic ROS-specific probe, the fluorescence of which is activated through H_2_O_2_ mediated specific boronate-to-phenol transition,^45,70–72^ was used to verify H_2_O_2_ in SGs. The signal significantly decreased with the addition of 1,6-hexanediol (Figure S5). These results verify that H_2_O_2_ is the source of electroactive species of SGs.

**Figure 3:**
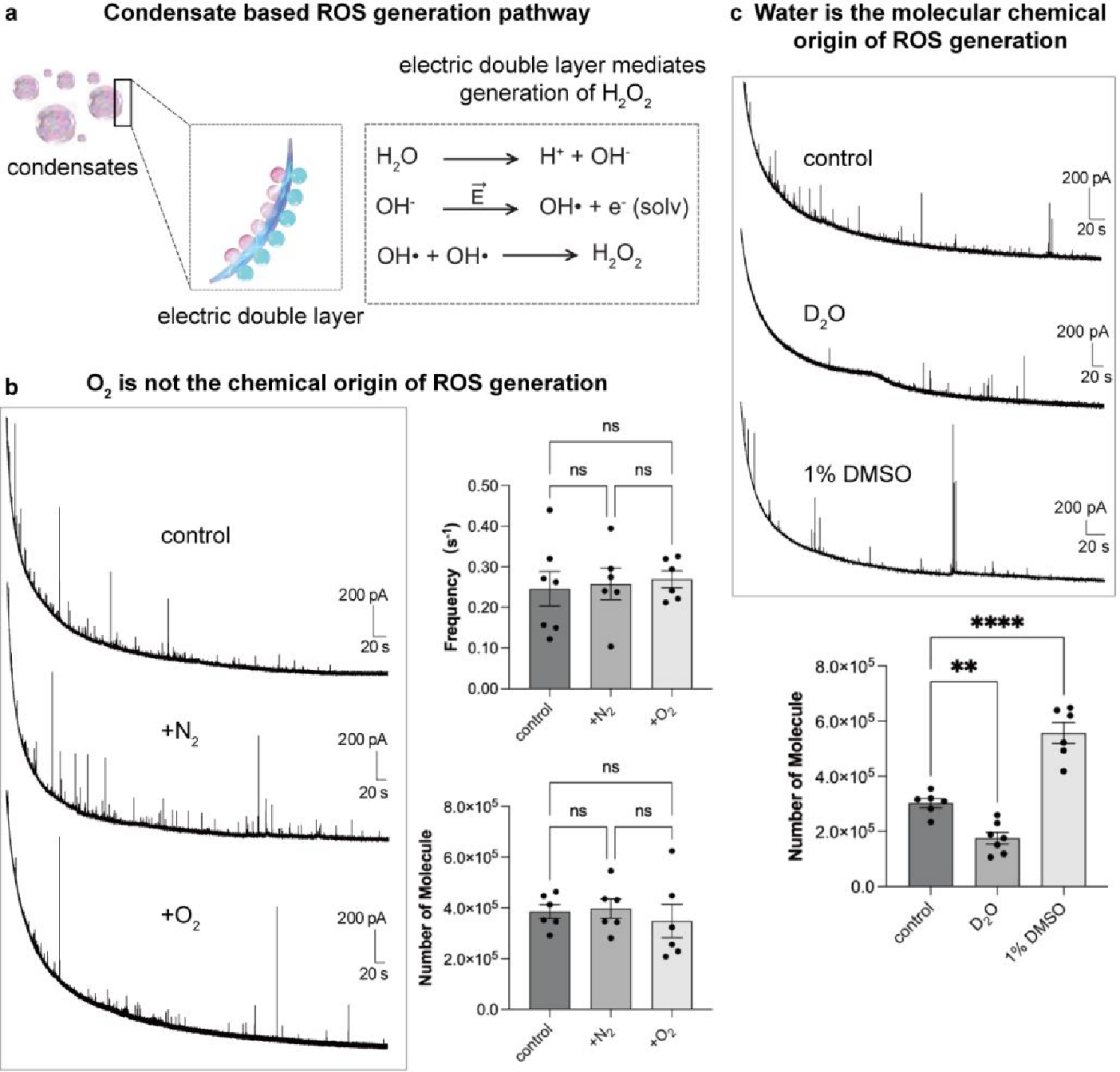
(a) Schematic mechanism of H_2_O_2_ produced in SG through interfacial redox chemistry. (b) Typical amperometry traces obtained by the nanosensors in ex-vivo separated SGs dispersed in normal, N_2_ or O_2_ purged lysis buffer. The potential applied at the nanosensors was +800 mV vs Ag/AgCl. Bar graphs show the statistical median for frequency of detected SG events and the amount of H_2_O_2_ included in SGs. Statistical data for each group: control, *n* = 6 for 591 SGs; +N_2_, *n* = 6 for 678 SGs; +O_2_, *n* = 6 for 598 SGs. (c) Typical amperometry traces obtained by the nanosensors in ex-vivo separated SGs dispersed in lysis buffer prepared by H_2_O (control) and D_2_O or by H_2_O with the addition of 1% DMSO. The potential applied to nanosensors was +800 mV vs Ag/AgCl. Bar graphs show the statistical median for frequency of detected SG events and the amount of H_2_O_2_ included in SGs. Statistical data for each group: control, *n* = 6 for 619 SGs; D_2_O, *n* = 7 for 410 SGs; 1% DMSO, *n* = 6 for 328 SGs. Data represent means ± SEM. One-way ANOVA on ranks: none, *p* > 0.5; **p < 0.01 and ****p < 0.0001.

We next reasoned that if ROS is generated through the interfacial electric field, then the solvent environment should regulate the interfacial electric field and determine the chemical origin of the ROS, thereby affecting the detectable ROS quantity. Thus, we first tested the ex-vivo separated SGs by incubating them either in N_2_ or O_2_ purged buffer solution. We did not observe significant differences in H_2_O_2_ quantity and frequency of the detected SGs between SGs in solutions before and after processing with N_2_ or O_2_ (Figure 3b). This observation suggests that solvated O_2_ is not the main chemical origin of the redox activities of SGs, implying that the production of H_2_O_2_ is not an enzymatic reaction originating from solvated O_2_. It is worth noting that the baseline current drop occurs for both amperometry traces obtained in living cells and ex-vivo separated SGs which is most likely from some form of background signals. However, it does not affect the spikes as these are not dependent on the background current (Figure S3).

To evaluate the role of water molecules in the production of H_2_O_2_, we examined SGs in a deuterium oxide-based solution. We observed that SGs in deuterium oxide-based solution produced 48% less H_2_O_2_ compared to those in water-based solution (Figure 3c). This observation suggests that dissociated OH from water molecules is the main source of the redox species observed at SGs. We next evaluated whether modulating the water activity in the solution could further change the redox capability of SGs. To this end, we added DMSO (1%) to the solution, thus altering the hydrogen bond network of water molecules^73^ and thereby increasing the quantity of free water molecules. We found that compared to the SG solution, adding 1% DMSO to the solution enhanced the production of H_2_O_2_ by 69% (Figure 3c). These experiments suggest a nonenzymatic pathway for the generation of redox species by SGs and identify that water molecules are the key source for the nonenzymatic pathway.

### Dynamic Generation of H_2_O_2_ in Ex-Vivo Separated SGs Modulated by Interfacial Electrochemical Activity

We then evaluated whether the ex-vivo separated SGs possess the basic electrochemical feature of condensates, the interfacial electric field, to mediate the promotion of redox reactions by visualizing the interfacial field by fluorescence assay.^21^ Specifically, a field-dependent fluorogneic DI-4-ANEPPS^74,75^ was applied to evaluate the existence of the field.^21,76^ Indeed, significant activation of the fluorescent signal was observed at the interface of SGs (Figure 4a and its fluorescent ratio decreased with the addition of 1,6-hexanediol (Figure S6), suggesting the existence of an interfacial electric field at SG.

**Figure 4:**
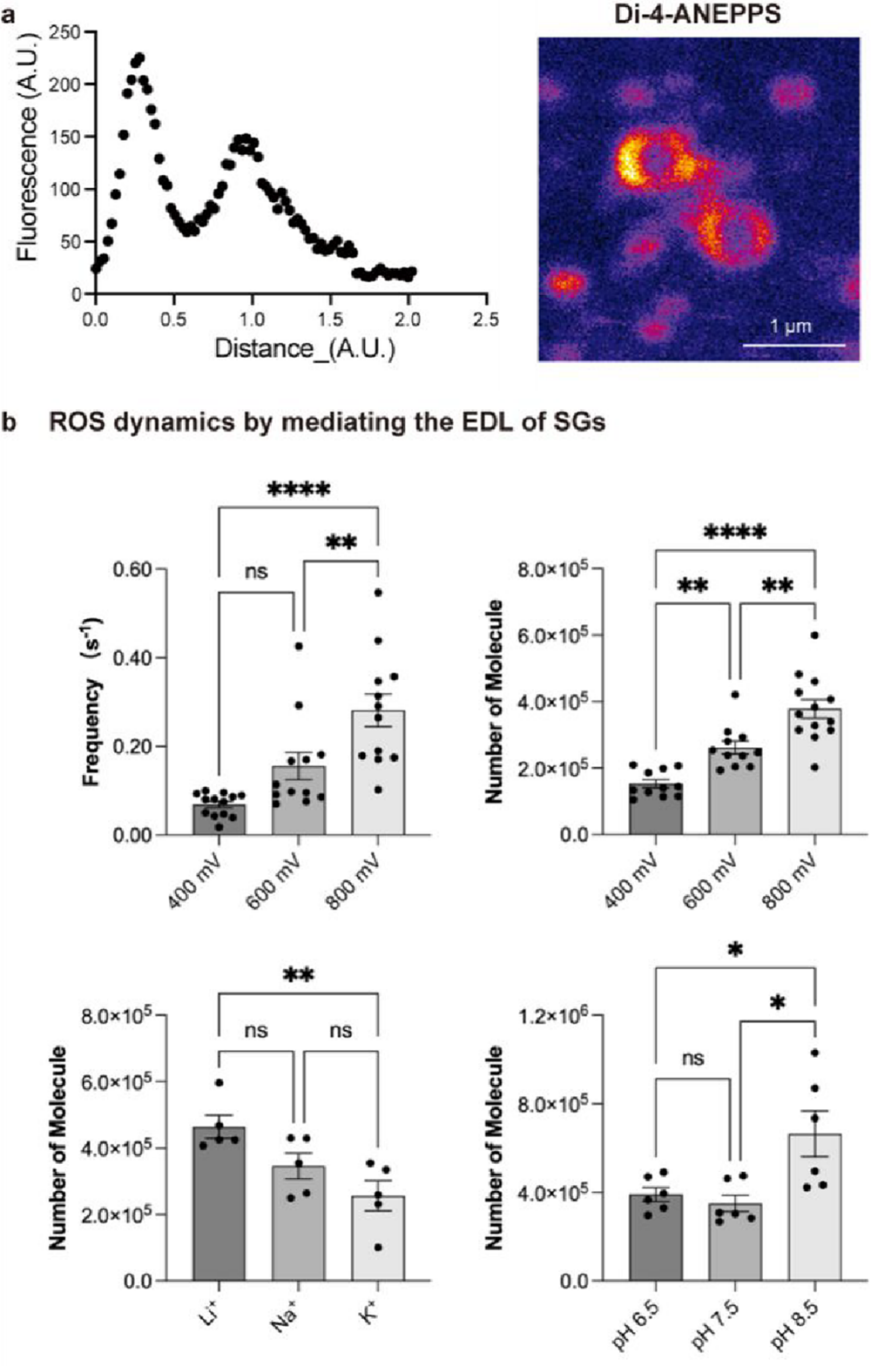
(a) Di-4-ANEPPS adsorbs at the SG interface and fluoresces upon adsorption. The graph on the right shows the distribution of di-4-ANEPPS fluorescence emission through a radial cross-section of the SG. (b) Bar graphs show the statistical median for frequency of detected SG events (top left panel) and the H_2_O_2_ number (top right panel) obtained at different potentials including +400 mV, +600 mV, and +800 mV vs Ag/AgCl, and the H_2_O_2_ amount obtained at +800 mV vs Ag/AgCl in SGs dispersed in lysis buffer prepared by Li^+^, Na^+^, or K^+^ (bottom left) and in lysis buffer with pH adjusted to 6.5, 7.5, or 8.5 (bottom right panel). Statistical data for each group: +400 mV, *n* = 13 for 361 SGs; +600 mV, *n* = 12 for 686 SGs; +800 mV, *n* = 12 for 1101 SGs; Li^+^, *n* = 5 for 263 SGs; Na^+^, *n* = 5 for 287 SGs; or K^+^, *n* = 5 for 249 SGs; pH 6.5, *n* = 6 for 345 SGs; pH 7.5, *n* = 6 for 415 SGs; pH 8.5, *n* = 6 for 397 SGs. Data represent means ± SEM. One-way ANOVA on ranks: none, *p* > 0.5; *, *p* < 0.05; **, *p* < 0.01; ****, *p* < 0.0001.

Inspired by the effect of surface charge of a solid on modulating the interfacial activity of contacted water droplets,^29,77^ we then probed the H_2_O_2_ in ex-vivo separated SGs at different electrode potentials (+400, +600, +800 mV vs Ag/AgCl), which are all sufficient to drive electrochemical oxidation of H_2_O_2_ at the nanosensor. As shown in Figure S7, SGs sequentially impact the electrode, and a series of oxidation spikes are observed at each of the three potentials. We found that the detection frequency is significantly higher, with potential increasing from +400 to +800 mV (Figure 4b, top left panel) and the amount of H_2_O_2_ demonstrated a similar electrode potential-dependent behavior (Figure 4b, top right panel). It is reported that when a positive potential is applied to an electrode, the positively charged electrode creates an electric field that extends into the solution, affecting the effective surface charges of charged particles by polarizing the surrounding electrolyte.^72^ This can interact with and modulate the interfacial electric field of SGs leading to the varied activity of redox reactions in single SGs.

We further evaluated the redox capability of SGs by dispersing the ex-vivo separated SGs into solutions containing different types of salts, Na^+^, K^+,^ and Li^+^ (Figure 4b, bottom left panel) and at different pH (Figure 4b, bottom right panel). The cations of these salts, due to their distinct lattice free energy and solvation structure,^78–81^ possess different capabilities to screen the electric double layer (EDL), thereby modulating the strength of the field across the EDL. Indeed, a significantly higher H_2_O_2_ number (4.64 × 10^5^) is obtained from SGs dispersed in solution containing Li^+^ than SGs dispersed in solutions containing K^+^ (2.56 × 10^5^ molecules) and Na^+^ (3.46 × 10^5^). Then we used different concentrations of sodium acetate to disperse ex-vivo SGs and found an increased number with the increase of Na^+^ (Figure S8 left). Similarly, a more basic solvent (pH = 8.5) promotes the generation of H_2_O_2_ at SGs significantly (6.64 × 10^5^) than a solvent at pH = 6.5 or 7.5 (3.91 × 10^5^ or 3.50 × 10^5^, respectively). Furthermore, we implemented an active strategy to modulate the interface of SGs, in which 1% sodium dodecyl sulfate (SDS), an anionic surfactant, was added to affect the interfacial electric field by organizing the interfacial ion alignment,^41^ leading to a 52% increase in the average number of H_2_O_2_ molecules observed per impact (Figure S8 right). In contrast, after adding CTAB, a cation surfactant, we did not observe a significant signal change. To further verify the change of H_2_O_2_ number caused by the solvent, we used a fluorogenic redox probe to track the H_2_O_2_ generation by SGs in lysis buffer prepared with Na^+^ or Li^+^. As shown in Figure S9, the fluorescent results were in accordance with those obtained by nanosensors, which reinforces the role of the surrounding environment in modulating the electrochemical activity of SGs.

### In Situ Analysis of ROS Dynamics Generated by SGs in Mammalian Cells

After understanding the process in vitro, we reevaluated whether the electrochemical features could affect the generation of H_2_O_2_ at SGs in living U2OS cells. We reasoned that by tuning the extracellular osmolarity, the cells would possess distinct ionic environments,^82^ thereby altering the capability of SGs to generate H_2_O_2_. To this end, we inserted the nanosensor into a single U2OS cell and measured ROS at +800 mV versus Ag/AgCl to limit the background signals. Then we used arsenite to trigger the formation of SGs in the same U2OS cells^64,65^ and continuously tracked the electrochemical signal in situ*.* Transient current peaks indicating the presence of H_2_O_2_ from collision events and the presence began to appear at approximately 13 min after adding 500 μM arsenite into the culture medium (Figure 5a). Through fluorescent microscopy, we also observed that SGs began to form 13–15 min in U2OS cells after 500 μM arsenite induction (Movie S2).^75,76^ This suggests that the emergence of the electrochemical signal coordinates with the formation of SGs. By altering the extracellular ionic concentrations, we found that an 86.2% higher number of H_2_O_2_ molecules is obtained in arsenite-stressed U2OS cells in hypertonic solution compared with isotonic solution (Figure 5b). Additionally, the frequency of detected SGs doubled at higher osmolality. It is worth noting that under fluorescent microscopy, we did not observe a significant change in the size and number of SGs in live arsenite-stressed cells after they were maintained in isotonic solution and hypertonic solution for 30 min (Movies S3, and S4). These data demonstrate that the intracellular ionic environment can tune the electrochemical activity of SGs, which suggests that the solvent electrochemical environments could be pivotal to define the electrochemical activities of SGs.

**Figure 5:**
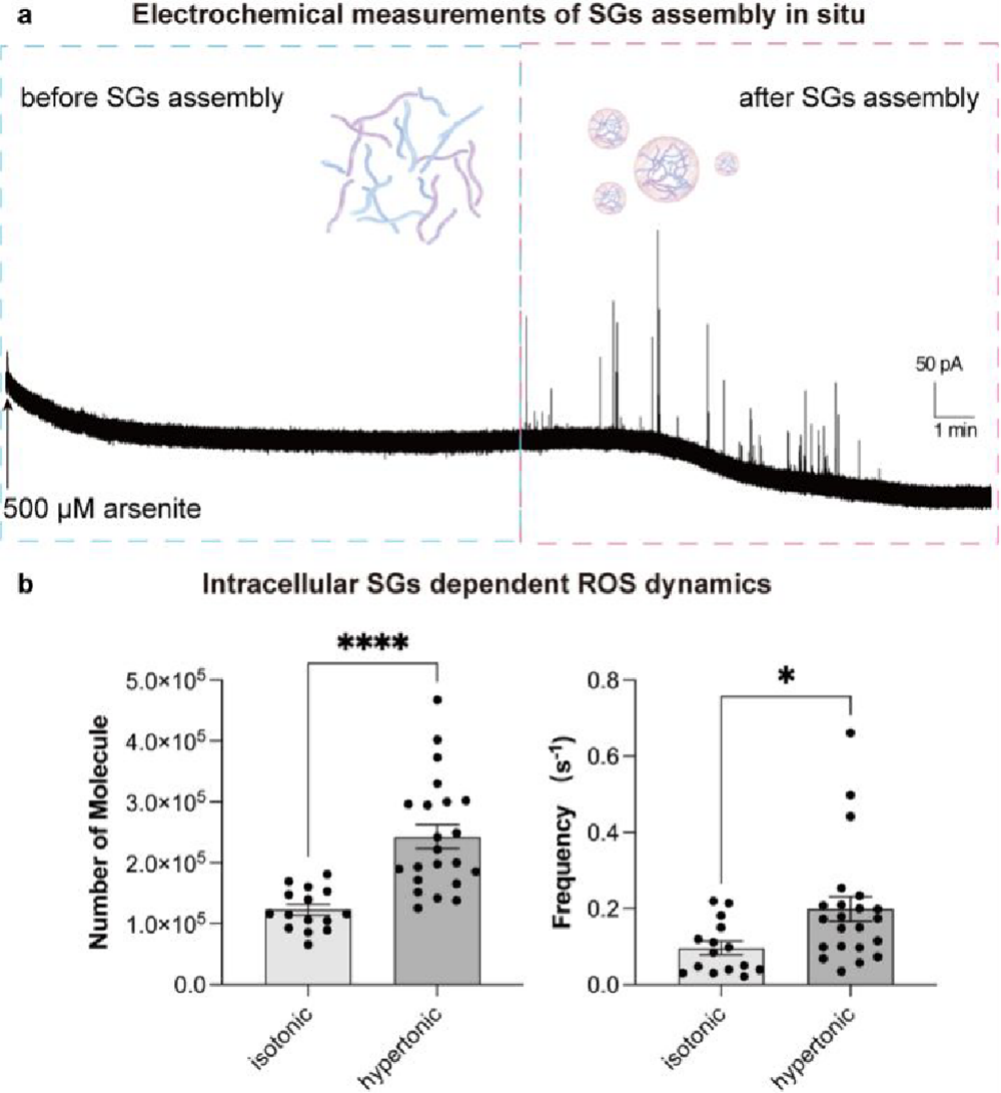
(a) Typical amperometry trace obtained by a nanosensor in a U2OS cell stressed by 500 μM arsenite to trigger the generation of SGs in situ. The potential applied at the nanosensor was +400 mV vs Ag/AgCl. (b) Bar graphs show the statistical median for frequency of detected SG events and the H_2_O_2_ number obtained in 200 μM arsenite-stressed U2OS cells subjected to isotonic solution (330 mOsm/kg) and hypertonic solution (730 mOsm/kg). The potential applied at the nanosensor was +400 mV vs Ag/AgCl. Statistical data for each group: isotonic, *n* = 15 for 452 SGs; hypertonic, *n* = 22 for 941 SGs. Data represent means ± SEM. Two-tailed Mann–Whitney rank-sum test; *, *p* < 0.05; ****, *p* < 0.0001.

A recent study^54^ showed that in an in vitro reconstitution experiment, condensates formed by G3BP1 and RNA, which are key components of native SGs, are able to promote the disulfide bond formation of TDP-43 proteins in a redox-dependent manner, leading to intracondensate demixing behavior. This implies that the formation of condensates can define an active redox environment. In our work, we implemented an electrochemical technique to examine the generation of ROS from ex-vivo separated native SGs. The number of H_2_O_2_ contained in ex-vivo separated SG is calculated to be 1.52 × 10^5^, and the median diameter of SGs is reported to be 273 nm^78^; thus, the concentration of H_2_O_2_ in native SGs is calculated to be 23.70 mM. This fundamental capability of native condensates in generating ROS provides a distinct pathway for cellular signaling and redox homeostasis.^79^

## Conclusions

We demonstrate the design and application of a simple collision-based electrochemical nanosensor to study the electrochemical activity of SGs via single-particle impact with the sensor. We provide evidence that native SGs produce ROS via water-dependent interfacial redox chemistry. This discovery has a potential impact on many aspects of our understanding of the conventional oxygen-dependent cellular pathways for the generation of ROS in cells.

Our data suggest, through both electrochemical and fluorescence measurements, that the oxidative peaks observed at SGs both in vitro and in living cells are due to H_2_O_2_ and are most likely generated via an electrochemical gradient across the interface of the SG and not via enzymatic processes in the SG. It is certainly possible that both occur, but the combined data with pH, potential dependence, ionic gradients, water dependence, and fluorescence measurements suggest that the main player is the inherent electrochemical activity at the SG–solution interface. Further work on identifying the composition dependency of such systems will expand our understanding on the molecular mechanisms driving such interface-dependent nonenzymatic redox activities.

In summary, we provide evidence for a nonenzymatic cellular pathway for the generation of reactive oxygen species at SGs with a distinct electrochemical mechanism. The nanosensing electrochemical approach developed here should be generally applicable to the study of the electrochemical activities of biomolecular condensates in living cells. Given the ubiquitous presence and significant role of condensates in diverse cellular processes coupled with our limited understanding of their electrochemical properties, our study introduces a novel paradigm at the interface of bionanotechnology and biomolecular condensates.

## Supplementary Material









## Notes

Experimental section and additional data and figures including cyclic voltammetry, fluorescence images, average number of events and the statistical median for number of molecules of events from the high and low frequency regions, typical amperometry traces, ROS-specific probe for the detection of ex-vivo separated SGs, and Di-4-ANEPPS assay (PDF)
Formation of SGs 13–15 min in U2OS cells after 500 μM arsenite induction (MP4)
No significant change in the size of SGs in live arsenite-stressed cells after they were maintained in isotonic solution and hypertonic solution for 30 min (MP4)
No significant change in the number of SGs in live arsenite-stressed cells after they were maintained in isotonic solution and hypertonic solution for 30 min (MP4)

## Abbreviations

SGs: Stress granules
ROS: Reactive oxygen species
NDs: neurodegenerative diseases
RNS: Reactive nitrogen species
SEM: Scanning Electron Microscope
