# Supplementary material for: Single-Entity Resolution Single-Cell Nanosensor Reveals Reactive Oxygen Species at Stress Granules Are Formed by Interfacial Redox Chemistry
Source: J Am Chem Soc. 2025 Jul 22;147(30):27020–9. doi: 10.1021/jacs.5c09338 (PMC12314916; doi:10.1021/jacs.5c09338)
Supplement: Supplementary file 1 [file ja5c09338_si_001.pdf]

# Single-entity resolution single-cell nanosensor reveals reactive oxygen species at stress granules are formed by interfacial redox chemistry

Hui Gu[a,b]*, Chaoyi Gu[b], Andre Du Toit[b], Wen Yu[c], Michael W. Chen[c], Heather L Struckman[c], Jonathan R Silva[c], Yifan Dai[c]*, Andrew G. Ewing[b]*.

[a]Department of Chemistry and Chemical Engineering, Hunan University of Science and Technology, Xiangtan, 411201, China.

[b]Department of Chemistry and Molecular Biology, University of Gothenburg, Gothenburg, 41390, Sweden.

[c]Department of Biomedical Engineering and Center for Biomolecular Condensates, Washington University in St. Louis, Saint Louis, 63130, USA.

## Table of Contents


1. EXPERIMENTAL

1.1. Fabrication of the electrochemical nanosensor

  1.1.1. Preparation of carbon nanotip electrodes

  1.1.2. Platinization of carbon nanotip electrodes

1.2. U2OS cell cultures

1.3. E*x-vivo* SGs from U2OS cells

1.4. Electrochemical measurements

  1.4.1. Electrochemical measurements of *ex-vivo* SGs

  1.4.2. Electrochemical measurements of SGs in U2OS cells

1.5. Fluorogenic PO-1 analysis of hydrogen peroxide

1.6. Confocal evaluation of interfacial electric field with DI-4-ANEPPS

1.7. Confocal imaging of live U2OS cells

1.8. Data analysis

2. FIGURES

Figure S1. Typical cyclic voltametric curve of electrochemical deposition to prepare electrochemical nanosensor, and its voltammetric oxidation of $H_2O_2$.

Figure S2. Representative images of U2OS cells before and after the cells were stressed by 200 μM arsenite.

Figure S3. Average number of events per cell for intracellular measurements of SGs by the nanosensor and the statistical median for number of molecules of events from the high (0-100 s) and low frequency (100-600 s) regions.

Figure S4. Typical amperometry traces obtained by electrochemical nanosensors of SGs before and after treated by1,6-hexanediol or by catalase.

Figure S5. PO-1 assay for the detection of $H_2O_2$ included in *ex-vivo* separated SGs.

Figure S6. Evaluation of interfacial potential of SGs via the Di-4-ANEPPS assay by fluorescence ratio by adding 6% 1,6-hexanediol.

Figure S7. Representative amperometry traces obtained in *ex-vivo* separated SGs with the electrochemical nanosensor at different potentials.

Figure S8. Comparison of the number of $H_2O_2$ molecules of SGs dispersed in lysis buffer with different $Na^+$ concentration or with addition of SDS or CTAB.

Figure S9. PL-1 assay for the detection of $H_2O_2$ in *ex-vivo* separated SGs dispersed in lysis buffer prepared by $Li^+$ or $Na^+$.

# 1. Experimental

## 1.1. Fabrication of the electrochemical nanosensor

### 1.1.1. Preparation of carbon nanotip electrodes

Fabrication of carbon nanotip electrodes was previously described.[1-3] First, a 5-μm diameter carbon fiber was aspirated into a borosilicate capillary (1.2 mm O.D., 0.69 mm I.D., Sutter Instrument Co., Novato, CA, U.S.A.). Then, the capillary was pulled in half with a micropipette puller (model PE-21, Narishige, Inc., Japan) to obtain two electrodes. Next, the fiber extending from the glass was cut to 100-150 μm length with a scalpel under a microscope. The electrodes were afterwards held on the edge of the blue part of a butane flame (Clas Ohlson, Sweden) to flame etch until a needle-sharp tip is about 50-200 nm tip diameter. The electrodes were then sealed by dipping the tip into epoxy solution (GA Lindberg ChemTech AB, Sweden), followed by drying in an oven at 100°C overnight.

### 1.1.2. Platinization of carbon nanotip electrodes

Platinizing solution was prepared by adding 1.5% $H_2PtCl_6$ and 80 μM lead (II) acetate to PBS. The nanotip electrodes were scanned from 0 to −500 mV vs Ag/AgCl reference electrode at a scan rate of 2 mV/s for 1 cycle using a CHI electrochemical analyzer (CH Instruments, Inc., Austin, TX, USA). The electrodes were then tested by cyclic voltammetry (vs Ag/AgCl, 100 mV/s) in a solution of $ONOO^-$ (pH=10.0, 10 μM), $H_2O_2$ (pH=7.4, 10 μM), NO• (pH=7.4, 10 μM)) and $NO_2^-$ (pH=7.4, 10 μM) in phosphate buffer saline (0.01 M). For NO• detection, 50 mM DEA-NONOate (Cayman Chemicals) was initially prepared in 0.01 M NaOH and conserved in an icebox for maximum 24 h before use. To initiate NO• release, minute aliquots of stock solution were rapidly mixed with PBS buffer (pH = 7.4). For $ONOO^-$ detection, $ONOO^-$ solutions were prepared by diluting alkaline stock solution (40 mM $NaONO_2$ stored at -80°C; Cayman Chemicals) with PBS buffer (pH = 10). Only those electrochemical nanosensors showing similar stable steady-state currents in Figure S1b were used for further electrochemical experiments.

## 1.2. U2OS and HEK293 cell cultures

High glucose Dulbecco's modified Eagle's medium (DMEM) supplemented with 10% fetal bovine serum (FBS), 1 % penicillin-streptomycin and 1 ug/mL puromycin was used to maintain Human bone osteosarcoma epithelial (U2OS) cells. The cells were grown on TC-treated T75 flasks (Sarstedt, Sweden) and cultured at 37°C in a 5 % $CO_2$ ,100% humidity incubator until the cells proliferated into a confluent monolayer.

Wild-type HEK293 cells were cultured in Dulbecco's modified Eagle medium with high glucose (ThermoFisher, 11965092) and 10% heat-inactivated fetal bovine serum (ThermoFisher, A3840101). Confluent cells were incubated with 0.5mM sodium

arsenite for 1 hr at 37°C.

### 1.3. E*x-vivo* SGs from U2OS cells

The SGs were separated according to a previously reported protocol.[4, 5] First, U2OS cells were stressed with 100 μM arsenite for 1 h and spun at 300 g for 10 min. Then, the obtained pellet was re-suspended in 1 mL of SG lysis buffer SG (50 mM Tris-HCl, 100 mM potassium acetate, 2 mM magnesium acetate, 0.5 mM DTT, 50 μg/mL heparin, 0.5 % NP-40, 1 complete mini EDTA-free protease inhibitor tablet) and snap frozen. The suspension was lysed by passing it through a 25 G 5/8 needle seven times on ice and then it was spun at 1000 g for 10 min. The supernatant containing SGs was centrifuged at 18,000 g for 20 min to obtain the SG pellet. This pellet was re-suspended in 1 mL of lysis buffer or modified lysis buffer followed by spin down again at 18,000 g for 20 min. All the centrifugation steps were performed at 4 °C. SGs isolated from HEK293 cells followed the same procedure with U2OS cell. Except for Figure S6, all SGs were seperated from U2OS cell.

### 1.4. Electrochemical measurements

All amperometric measurements were performed using a two-electrode electrochemical system in a well-grounded Faraday cage. The working potential was +800 mV vs. an Ag/AgCl reference electrode (Scanbur, Sweden), except where there is additional description, under the control of an Axopatch 200B potentiostat (Molecular Devices, Sunnyvale, CA). The output was digitized at 10 kHz and filtered at 2 kHz using a 4-pole Bessel filter.

1.4.1. Electrochemical measurements of *ex-vivo* SGs

Before electrochemical measurements, the SG pellets were re-suspended in 100 μL of lysis buffer or modified lysis buffer. Modified lysis buffers were prepared here to investigate how solvent modulates the interaction with SGs, including $K^+$, $Na^+$, $Li^+$ series lysis buffers (50 mM Tris-HCl, 100 mM potassium acetate/sodium acetate/Lithium acetate, 0.5 mM DTT, 50 μg/mL heparin, 0.5 % NP-40, 1 complete mini EDTA-free protease inhibitor tablet), different pH series lysis buffers with pH adjusted to 6.5, 7.5 or 8.5 by sodium hydroxide, different concentration of $Na^+$ series lysis buffers (50 mM Tris-HCl, 10 mM, 150 mM, 500 mM, 1000 mM sodium acetate (NaAc), 0.5 mM DTT, 50 μg/mL heparin, 0.5 % NP-40, 1 complete mini EDTA-free protease inhibitor tablet), deutroxide ($D_2O$) lysis buffer (regular lysis recipe with $D_2O$ as solvent), 1% dimethylsulfoxide (regular lysis buffer by adding 1% DMSO). For the SDS, 1,6-hexanediol and catalase, 1% sodium dodecyl sulfate (SDS), 1% cetyltrimethyl ammonium bromide (CTAB). 6% 1,6-hexanediol, or 2.5% catalase was added before electrochemical experiments). For $N_2$ or $O_2$, regular lysis buffers were purged with $N_2$ or $O_2$ at 25 mL/s for 30 min to remove or saturate the dissolved $O_2$ before they were used to disperse *ex-vivo* separated SGs.

1.4.2 Electrochemical measurements of SGs in U2OS cells

U2OS cells were observed under an inverted microscope (IX81, Olympus) with 10x and 40x objectives. The nano-electrochemical sensor was controlled precisely with a patch-clamp micromanipulator (PCS-5000, Burleigh Instruments, Inc., USA) to be first placed on the top of a U2OS cell followed by popping through the membrane of this U2OS cell while the current was recorded. Current transients were recorded and digitized using a Digidata1440A (Molecular Devices) and digitized at 10 kHz and filtered at 2 kHz using a 4-pole Bessel filter. To stimulate SG assembly, U2OS cells were treated with 200 μM arsenite for 1 h. For 1,6-hexanediol and catalase treatment, U2OS cells were treated by 200 μM arsenite for 1 h followed by 6% 1,6-hexanediol or 2000U/mL catalase-polyethylene glycol for 10 min. Before electrochemical experiments, the cells were stabilized in isotonic saline solution (150 mM NaCl, 5 mM KCl, 1.2 mM $MgCl_2$, 2 mM $CaCl_2$, 5 mM glucose and 10 mM HEPES, pH 7.4). U2OS cells were treated by 200 μM arsenite and then the medium was changed to isotonic solution (150 mM NaCl, 5 mM KCl, 1.2 mM $MgCl_2$, 2 mM $MgCl_2$,5 mM glucose, 10 mM HEPES, pH 7.4) with an osmolality of 330 mOsm/kg or to hypertonic solution with an osmolality of 730 mOsm/kg (adjusting isotonic solution by adding NaCl). For *in situ* electrochemical measurements of SGs in U2OS cells, when U2OS cells in isotonic saline solution had 500 uM arsenite added to trigger assembly of SGs *in situ*, an electrochemical nanosensor was placed in the U2OS cell to perform simultaneous current recording.

### 1.5. Fluorogenic PO-1 analysis of hydrogen peroxide

Fluorogenic Peroxy Lucifer 1 (PL-1) and Peroxy Lucifer 1 (PL-1) were used for the analysis of $H_2O_2$ production from separated stress granules. 50 μM PO-1 was added to the *ex-vivo* seperated SG sample with or without addition of 6% 1, 6-hexanediol (10 min). The samples were incubated at room temperature for 30 min before proceeding with analysis using Multimode Tecan plate reader. For PO-1, the excitation was set at 540 nm and the detector was set at 560 nm with a bandwidth of 10 nm. For PL-1, The sample was incubated at room temperature for 60 min before proceeding with the confocal microscopy with LEICA STELLARIS 8 FALCON. The excitation of PL-1 was set at 405 nm and and emission detector range (450-490 nm and 530-570 nm). DIC images were taken under the same excitation wavelength setup of the fluorescent images with a voltage at around 300 V-350 V.

### 1.6. Confocal evaluation of interfacial electric field with DI-4-ANEPPS

DI-4-ANEPPS was used for evaluation of the interfacial electric field of stress granules. 10 μM DI-4-ANEPPS was added into separated stress granules sample. The sample was incubated at room temperature for 30 min before proceeding with the confocal microscopy with LEICA STELLARIS 8 FALCON. For ratiometric analysis, two HYD detectors were set at 535–545 and 610–640 nm with an excitation wavelength at 470 nm. The images were processed by ImageJ.

### 1.7. Confocal imaging of live U2OS cells

SG formation was visualized in U2OS cells stably expressing G3BP1-GFP using an Abberior confocal microscope (Abberior, Germany). Imaging was performed using preset configurations for DAPI and GFP. For excitation, 405 nm and 485 nm lasers were used to detect DAPI and GFP, respectively. Emission was collected in the ranges of 415–475 nm for DAPI and 495–571 nm for GFP. The field of view was set to 80 µm with a pixel size of 100 nm. Laser power, dwell time, pin hole and the number of line scans were optimized to achieve a high signal-to-noise ratio. For live-cell imaging, an on-stage incubator was used to maintain cells at 37°C. Time-lapse imaging was conducted over a 0.5 h period with images acquired every 30 secs using the same DAPI and GFP settings.

### 1.8. Data analysis

All amperometry traces were converted into txt files by use of Matlab (The MathWorks, Inc.) and then processed by Igor Pro 6.22 o (Wavemetrics, Lake Oswego, OR). The traces were carefully inspected after peak detection and false positives were manually rejected. The data for number of molecules from single SGs, for $t_{1/2}$, and for frequency of the events were each pooled, and the medians of the data were calculated for each experimental condition. To compare between different conditions, means of medians of the above parameters were calculated, groups were statistically analyzed with the One-way ANOVA (for three or more than three groups) or Mann–Whitney rank sum test (unpaired and two-tailed, for two groups) using Prism 10 (GraphPad, La Jolla, CA) (****, $p < 0.0001$; ***, $p < 0.001$; **, $p < 0.01$; *, $p < 0.05$; none, $p>0.5$).

## 2. Figures

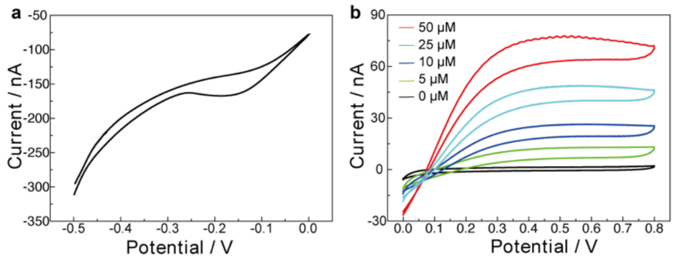


**Figure S1.** a) Typical cyclic voltametric curve of a carbon nanotip electrode during electrochemical deposition of a platinum-petal structure on the surface. Parameters: potential range from 0 to −500 mV vs Ag/AgCl, scan rate 2 mV/s. b) Voltammetric oxidation of different concentration of $H_2O_2$ (5, 10, 25, 50 μM) in PBS, scan rate 0.1 V/s.

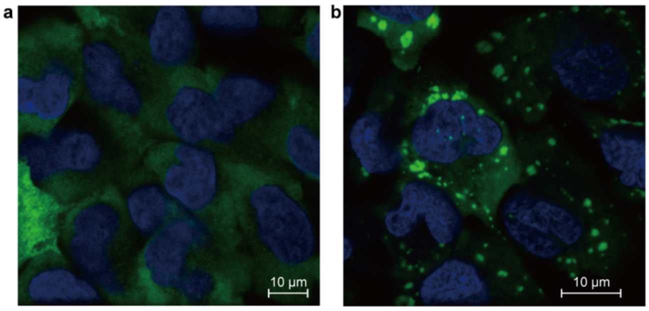


**Figure S2.** Representative images of U2OS cells stably expressing G3BP1-GFP (green) stained for nucleus with DAPI (blue) showing SG (green agrigates) before (a) and after (b) the cells were stressed by 200 μM arsenite.

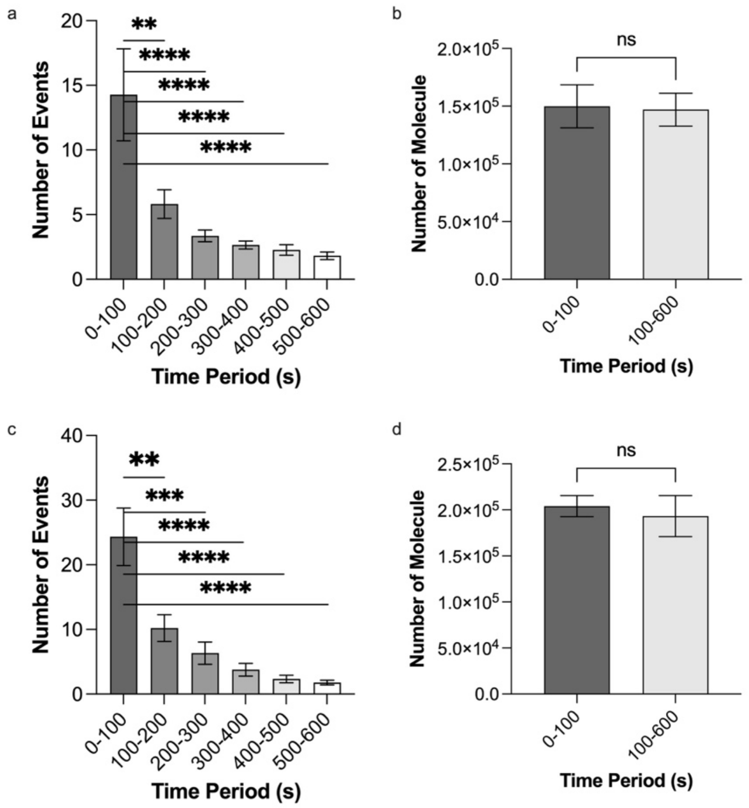


**Figure S3.** Average number of events per cell for intracellular measurements of SGs by the nanosensor at 400 mV (a) and 800 mV (c) vs. Ag/AgCl. Bar graphs show the statistical median for number of molecules of events from the high (0-100 s) and low frequency (100-600 s) regions. The number of cells used for 400 mV was 11 (332 SGs) and for 800 mV was 9 (439 SGs). Data represent means ± SEM. Data represent means ± SEM. One-way ANOVA on ranks: ns $p>0.5$; ** $p < 0.01$; *** $p < 0.001$ and **** $p < 0.0001$.

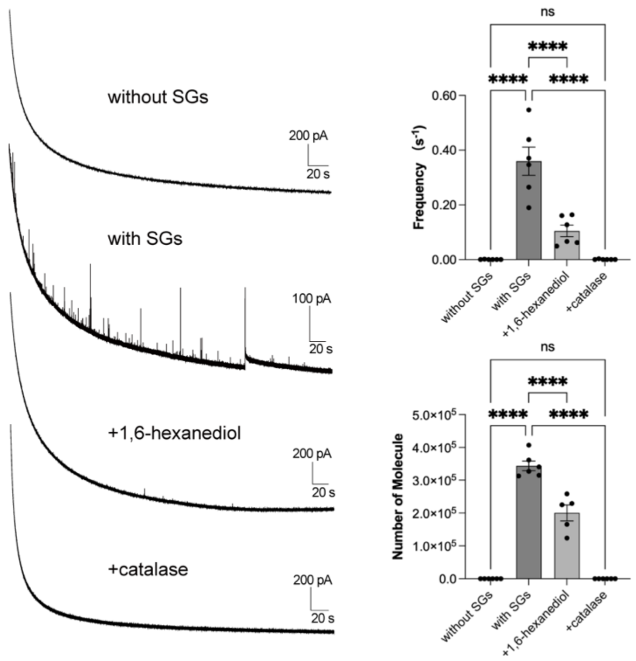


**Figure S4.** Typical amperometry traces obtained by electrochemical nanosensors in lysis buffer without or with SGs, or with SGs after treated by 6% 1,6-hexanediol or by 2.5% catalase for 10 min. The potential applied at the nanosensors was +800 mV vs. Ag/AgCl. Every spike represents the oxidation of $H_2O_2$ at a single SG. Bar graphs show the statistical median for the frequency of detected SG events and the number of $H_2O_2$ included in SGs. Statistical data for each group: control, n=6 for 749 SGs; 1,6-hexanediol, n=6 for 228 SGs; catalase, n=6 for 3 spikes.

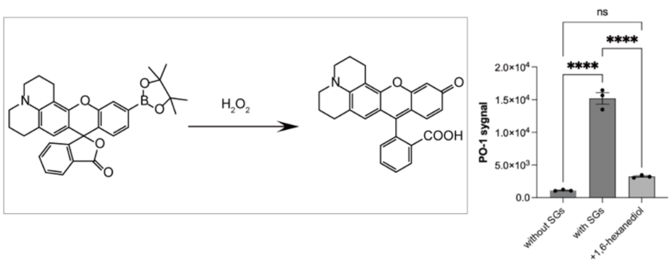


**Figure S5.** PO-1 assay for the detection of $H_2O_2$ and its application in detection of $H_2O_2$ included in *ex-vivo* separated SGs. Bar graph shows the statistical median for PO-1 fluorescence signal obtained in lysis buffer without or with SGs and with SGs followed by treatment by 6% 1,6-hexanediol for 10 min. Data represent means ± SEM. One-way ANOVA on ranks: *, $p<0.05$, **$p < 0.01$ and ****$p < 0.0001$.

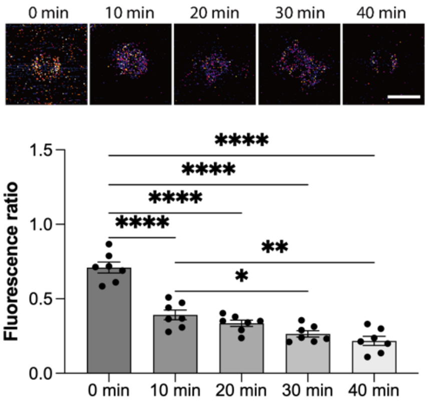


**Figure S6.** Evaluation of interfacial potential of *ex-vivo* separated SGs via the Di-4-ANEPPS assay by fluorescence ratio by adding 6% 1,6-hexanediol. Scale bar is 2 μm. Data represent means ± SEM. One-way ANOVA on ranks: *, $p<0.05$, **$p < 0.01$ and ****$p < 0.0001$.

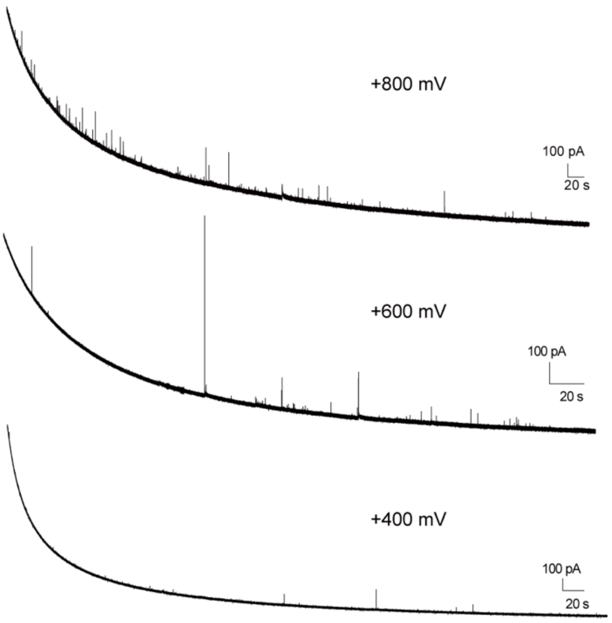


**Figure S7.** Representative amperometry traces obtained in *ex-vivo* separated SGs with the electrochemical nanosensor at +400 mV, +600 mV and 800 mV vs. Ag/AgCl.

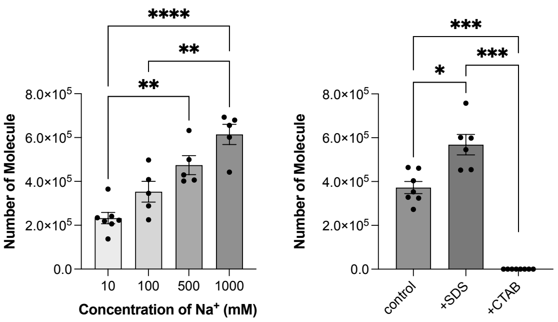


**Figure S8.** Comparison of the number of $H_2O_2$ molecules obtained at +800 mV vs. Ag/AgCl in SGs dispersed in lysis buffer with different $Na^+$ concentration (left panel) or with addition of 1% SDS or CTAB (right panel). Statistical data for each group: control, n=7 for 506 SGs; SDS, n=6 for 315 SGs; CTAB, n=7 for 0 SGs; $Na^+$ 10 mM, n=7 for 359; 100 mM, n=7 for 484; 500 mM, n=5 for 757; 1000 mM, n=5 for 284. Data represent means ± SEM. One-way ANOVA on ranks: *, $p<0.05$, **$p < 0.01$ and ****$p < 0.0001$.

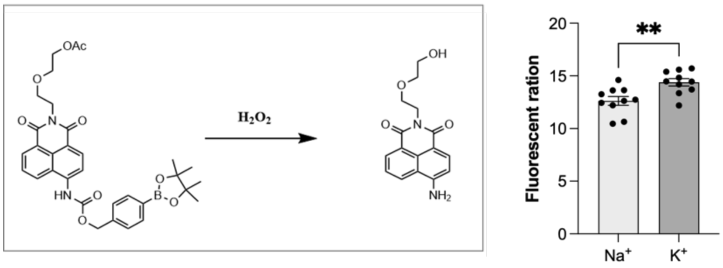


**Figure S9.** PL-1 assay for the detection of $H_2O_2$ and its application in detection of $H_2O_2$ included in *ex-vivo* separated SGs dispersed in lysis buffer prepared by $Na^+$ or $Li^+$. Data represent means ± SEM. One-way ANOVA on ranks: $**p < 0.01$.

References

(1) Strein, T. G.; Ewing, A. G. Characterization of submicron-sized carbon electrodes insulated with a phenol-allylphenol copolymer. *Anal. Chem.* **1992**, *64* (13), 1368-1373.

(2) Kawagoe, K. T.; Jankowski, J. A.; Wightman, R. M. Etched carbon-fiber electrodes as amperometric detectors of catecholamine secretion from isolated biological cells. *Anal. Chem.* **1991**, *63* (15), 1589-1594.

(3) Strand, A. M.; Venton, B. J. Flame Etching Enhances the Sensitivity of Carbon-Fiber Microelectrodes. *Anal. Chem.* **2008**, *80* (10), 3708-3715.

(4) Brocard, M.; Iadevaia, V.; Klein, P.; Hall, B.; Lewis, G.; Lu, J.; Burke, J.; Willcocks, M. M.; Parker, R.; Goodfellow, I. G.; et al. Norovirus infection results in eIF2alpha independent host translation shut-off and remodels the G3BP1 interactome evading stress granule formation. *PLoS Pathog* **2020**, *16* (1), e1008250.

(5) Hu, K.; Relton, E.; Locker, N.; Phan, N. T. N.; Ewing, A. G. Electrochemical Measurements Reveal Reactive Oxygen Species in Stress Granules. *Angew. Chem. Int. Ed.* **2021**, *60* (28), 15302-15306.
